# Optimized YOLOv11m for real-time high-speed railway catenary defect detection

**DOI:** 10.1038/s41598-025-29172-2

**Published:** 2025-12-13

**Authors:** Tao Jin, Zhijun Shen, Haowen Geng

**Affiliations:** 1 School of Computer and Information Engineering, Fuyang Normal University, Fuyang, 236037 Anhui People’s Republic of China; 2 Anhui Engineering Research Center for Intelligent Computing and Information Innovation, Fuyang Normal University, Fuyang, 236037 Anhui People’s Republic of China

**Keywords:** High-speed railway, Lightweight, YOLOv11m, Catenary, Defect detection, Mathematics and computing, Computational science, Computer science, Information technology

## Abstract

Real-time defect detection of high-speed railway catenary components remains challenging due to the prevalence of small-sized parts (e.g., cotter pins, fasteners) and the computational constraints of deployment platforms. While existing YOLO-based models offer a balance between speed and accuracy, they often struggle with small object detection and suffer from high computational costs. To address these limitations, this paper proposes an optimized YOLOv11m model, termed MSIM-YOLOv11m, which integrates three novel modules: large separable kernel attention (LSKA) for enhanced feature extraction, bidirectional feature pyramid network (BiFPN) for efficient multi-scale fusion, and adaptive kernel convolution (AKConv) for flexible feature learning. Experimental results on a dedicated catenary dataset show that the proposed model achieves a mAP50-95 of 78.3% and a small-target AP of 64.7%, while reducing computational cost by 50.5% compared to YOLOv9m. The model provides a lightweight and accurate solution suitable for real-time inspection applications.The code has been uploaded to https://github.com/1748125472/MSIM-Yolov11m/tree/master.

## Introduction

The catenary system is the only way for rail transit trains to obtain electric energy. Due to the long-term influence of the train’s own jitter and environmental factors, all kinds of parts in the catenary system, such as insulators, hanging strings, cotter pins, etc., are prone to defects, and due to the small size of some parts, it is difficult to be found in time once the defects occur^1^. Therefore, how to efficiently and accurately detect the defects of catenary parts is of great practical significance to ensure the operation safety of rail transit trains. At present, catenary defect detection has developed in the direction of intelligence, and catenary defect detection method based on deep learning has become the mainstream detection method.

In recent years, the operating mileage of high-speed electrified railways has grown rapidly, and the use of catenary insulation equipment has increased year by year. As an important equipment for electrical insulation and power support, the insulation performance of wrist arm insulator will have a huge impact on the safe operation of electrified railways^2–5^. Due to the unique nature of the application environment, the surface of the device is prone to visible damage and requires long-term monitoring to ensure safety^6–9^. At present, the general way of catenary detection is to use catenary detection vehicle to carry out full-line inspection, and combine manual fixed-point maintenance, but due to the problem of quantity, accuracy and efficiency, it is urgent to apply efficient intelligent image recognition technology to improve the level of monitoring and detection. At present, there are many studies on the use of deep learning to achieve object detection for high-speed rail catenary components, and the main research is to improve the image recognition rate. Object detection schemes based on deep learning can be roughly divided into two categories: two-stage detection schemes and single-stage detection schemes.

The two-stage detection protocol mainly includes R-CNN^10^, Fast RCNN^11^ and Faster R-CNN^12^, etc.Li Changjiang et al. designed a method based on Faster RCNN secondary cascade to complete the step-by-step positioning of the high-speed rail catenary support device, so as to locate the equipotential line, and then classify the fault, and the accuracy of identification was improved^13^. According to the three factors that affect the positioning of the screw of the diagonal brace sleeve, Wang Liyou improved the Faster R-CNN, and proposed a new image recognition method, which effectively improved the positioning accuracy of the screw^14^.

The single-stage detection scheme mainly includes SSD^15^ and YOLO^16–18^ series models. Peng Hao designed an improved version of the model based on YOLOv5^19^, it is possible to inspect insulators with small sizes and insulators with a high degree of overlap. Song et al.^20^ proposed an insulator defect detection algorithm based on Flexible YOLOv7, which integrates the attention mechanism in the process of feature extraction, reduces the parameter redundancy in the model training process by using an efficient SPPCSPC^21^ structure, and introduces the E-IOU loss function to focus on high-quality anchors^22^. The detection accuracy of the original model was improved, the extraction performance of dense targets, occlusions, and small target feature regions was enhanced, and the positioning and detection accuracy were optimized. In Ref^23^ a multi-scale dense convolutional network based on multi-scale feature fusion (MSD2Net) was proposed, and a multi-scale feature fusion network based on deconvolution and multi-branch detection was proposed to solve the problem of poor insulator recognition. In Ref^24^, a rod insulator detection model was constructed based on the deformable part model and latent SVM (Support Vector Machine), and the sub-images of different devices were extracted from the original image, and the rod insulators were identified and detected from them. In Ref^25^, an automatic fault diagnosis system was proposed, which can effectively identify loose strand defects of wires and eliminate the possibility of potential faults through an improved feature extraction network and an image segmentation method based on MRF (Markov random field).

While transformer-based architectures (e.g., EAPT^26^) show promise in feature extraction, their computational complexity limits real-time deployment. Similarly, NHBS-Net’s attention pyramid design improves segmentation but lacks adaptability to dynamic railway environments^27^. Existing methods struggle with small components (e.g., cotter pins) due to limited feature fusion strategies and redundant parameters. And recent advances like SES-YOLOv5^28^ enhance small object detection through graphics-oriented optimization, its reliance on single-scale feature extraction hinders performance in multi-component railway scenes. Similarly, detail-enhanced lightweight networks^29^ improve aerial image analysis but lack adaptive mechanisms for dynamic-scale targets like fasteners and insulators.

While the aforementioned studies have effectively realized the defect detection task of high-speed rail catenary parts through deep learning methods, several challenges remain: (1) Limited feature fusion strategies in existing methods lead to poor detection accuracy for small-volume parts such as cotter pins; (2) Many models contain parameter redundancies that hinder real-time deployment; (3) Most approaches lack adaptive mechanisms for handling dynamic-scale targets in complex railway environments.

To address these limitations, this paper proposes an optimized YOLOv11m-based detection framework with the following contributions:

1. We propose MSIM-YOLOv11m, a novel and efficient detector that integrates LSKA, BiFPN, and AKConv in a cohesive manner, specifically designed for the challenges in high-speed railway catenary defect detection, such as small targets, multi-scale components, and the need for computational efficiency.
2. We demonstrate how the LSKA module, with its large separable kernel attention, enhances the feature extraction for small targets, while the AKConv module adapts to the irregular shapes of catenary components. Combined with the BiFPN for multi-scale feature fusion, our model achieves a significant improvement in detecting small and complex catenary parts.
3. Our model achieves a superior balance between accuracy and computational cost, attaining a mAP50-95 of 78.3% while reducing the FLOPs by 50.5% compared to YOLOv9m, making it more suitable for real-time applications.
4. We build a dedicated dataset for high-speed railway catenary components and conduct comprehensive experiments, including ablation studies and comparisons with state-of-the-art detectors, to validate the effectiveness of our approach.

## YOLOv11 detection model framework

**Fig. 1:**
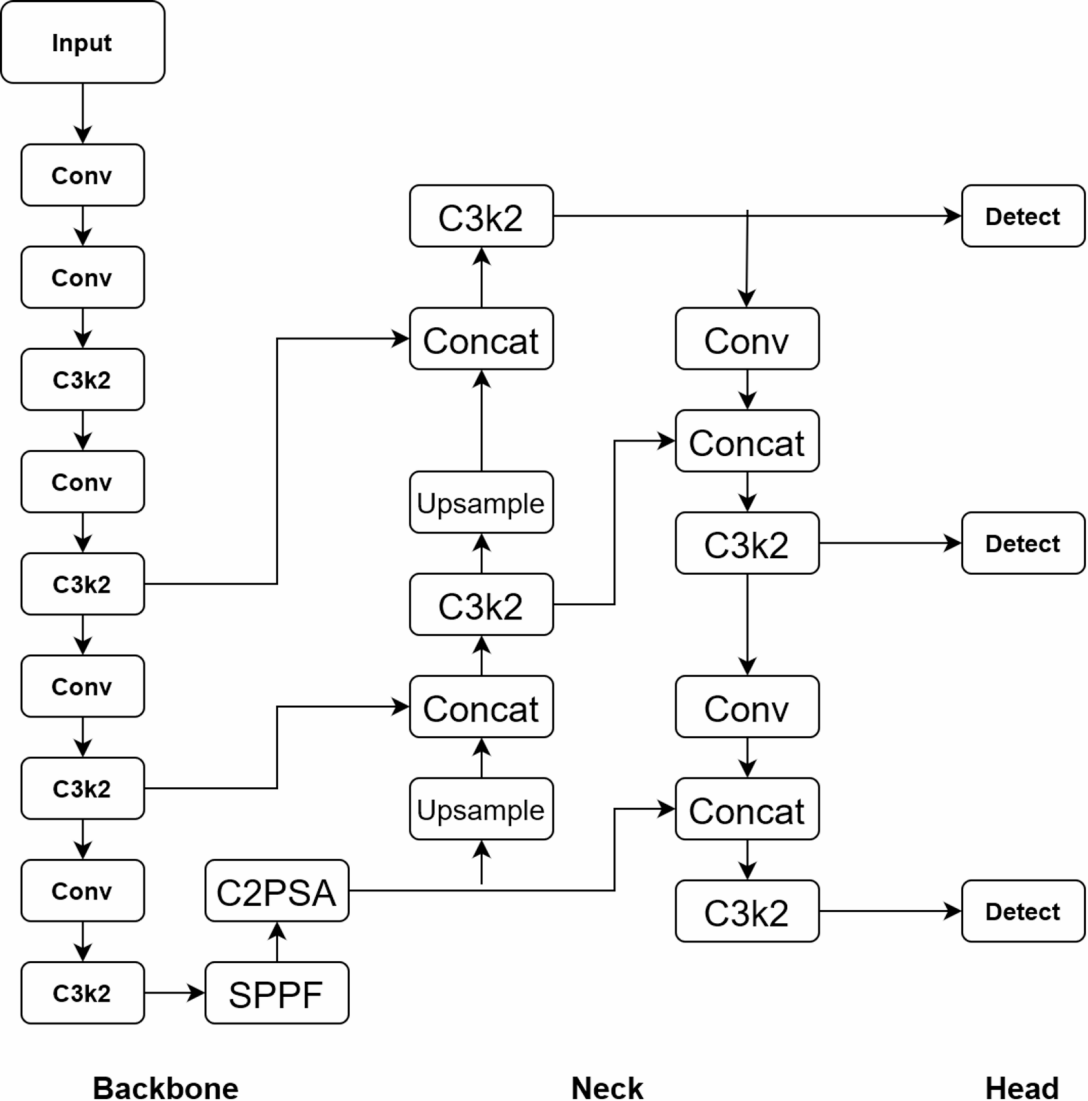
YOLOv11 model process framework.

The network structure of the YOLOv11 detection model is mainly divided into three parts: the backbone, the neck, and the head, as shown in Fig. 1 show. YOLOv11 utilizes CIoU Loss as the bounding box loss function, which considers overlap, center distance, and aspect ratio consistency for improved localization.The backbone of YOLOv11 adopts an improved backbone architecture, introducing C3K2 blocks to replace the C2F blocks in previous versions. The C3K2 block is actually converted from the C2F module, and when the c3k parameter is FALSE, the C3K2 module is the C2F module. When c3k is true, the bottleneck module is replaced with the C3 module. This improvement improves the computational efficiency and enhances the feature extraction ability. In the neck structure, YOLOv11 incorporates the C2PSA module. C2PSA is an extension of the C2F module, which incorporates PSA (Pointwise Spatial Attention) blocks to enhance feature extraction and attention mechanisms. By introducing PSA blocks in the standard C2F module, C2PSA implements a more powerful attention mechanism, which improves the model’s ability to capture important features. YOLOv11 uses multiple C3K2 blocks and CBS layers in the detection head section to further refine the feature map, and finally output bounding boxes and category labels. In addition, YOLOv11 also uses deep separable convolutions on the cls branch of the head section to reduce redundant computation and improve efficiency. The adaptive anchor frame mechanism can automatically optimize the anchor frame configuration on different datasets to improve the detection accuracy.

The YOLOv11m on which this article is based is a medium-sized model in the YOLOv11 series, compared with other versions of YOLOv11 (such as Nano, Small, Large, Extra-Large, etc.), its advantage lies in the ability to achieve a balance between accuracy and speed, YOLOv11m provides relatively fast processing speed while maintaining high accuracy, and compared to the Large and Extra-Large versions, YOLOv11m is more modest in terms of resource usage, it does not require excessive computing resources and storage space, while still being able to provide satisfactory performance.

## MSIM-Yolov11m model

### LSKA attention mechanism

**Fig. 2:**
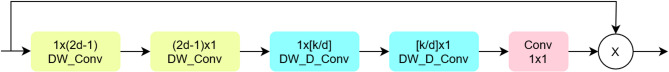
LSKA structure diagram.

LSKA (large separable kernel attention)^30^ The core principle of the attention module is to decompose the two-dimensional convolutional kernel into concatenated horizontal and vertical one-dimensional convolution kernels. This decomposition method makes it possible to use deep convolutional layers with large convolutional kernels directly in the attention module without the need for additional modules. LSKA implementation steps: Decompose the original 2D convolution kernel into two 1D convolution kernels, one for the horizontal direction (1xK) and the other for the vertical direction (Kx1), as shown in Fig. 2.This decomposition significantly reduces computational complexity and memory usage. The decomposed 1D convolution kernel is then cascaded to simulate the effect of the original 2D convolution kernel. Through cascade, the LSKA is able to capture both local and global feature information. After the cascade operation, the LSKA also incorporates the attention mechanism to further improve the representation ability of the model by weighting the importance of different feature channels.

LSKA replaces the standard attention layer in C2PSA, utilizing separable 1D convolution to reduce FLOP while enhancing the model’s feature extraction capabilities for more accurate detection results and good results in small object detection.

### Bidirectional characteristic pyramid network

**Fig. 3:**
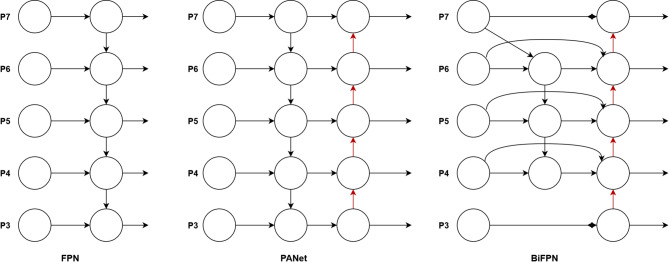
Comparison between BiFPN and other pyramid network structure.

Bidirectional Feature Pyramid Network (BiFPN)^31^ is an efficient multi-scale feature fusion network which is optimized on the basis of traditional Feature Pyramid Network (FPN).The main principle is that BiFPN allows features to be fused in both top-down and bottom-up directions, so as to combine features of different scales more effectively. By adding weights to each input feature, the feature fusion process is optimized, so that the network can pay more attention to features with larger amount of information. Cross-scale connections are optimized by removing nodes with only one input edge, adding extra edges between input and output nodes at the same level, and treating each bidirectional path as a feature network layer. Figure 3 shows the comparison of BiFPN results with other pyramid networks.

In our implementation, BiFPN integrates features from P3 to P7 levels (with strides of 8, 16, 32, 64, and 128 pixels respectively) extracted from the backbone network. Each bidirectional path is implemented as a repeatable block structure that enables efficient information exchange across scales.

FPN (Feature Pyramid Network) introduces a top-down path to fuse multi-scale features from Layer 3 to Layer 7 (P3–P7). PANet adds an additional bottom-up path to FPN. BiFPN improves the trade-off between accuracy and efficiency through efficient bidirectional cross-scale connections and repeatable block structures. It can be seen that BiFPN allows the bidirectional flow of feature information between different scales through the bidirectional path, and this bidirectional flow can be regarded as an effective information exchange between different scales. Such a design aims to improve the efficiency and effectiveness of feature fusion by enhancing the bidirectional flow of features, thereby improving the performance of object detection.

### AKConv variable kernel convolution

**Fig. 4:**
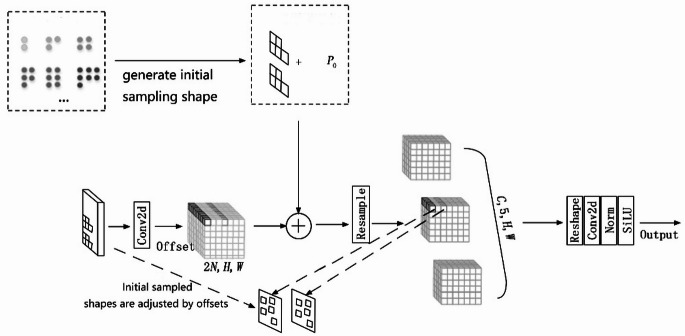
AKConv structure diagram.

AKConv(Alterable Kernel Convolution)^32^ The core idea is to provide the convolutional kernel with any number of parameters and any sample shape. This enables AKConv to extract features using any number of parameters, which is not implemented in standard and deformable convolution. This flexibility allows AKConv to better adapt to targets with changing shapes and sizes, improving the accuracy and efficiency of feature extraction. Traditional convolutional kernels usually have a fixed size and shape, such as a 3 × 3 or 5 × 5 square network. The core principle of AKConv is to allow the convolutional kernel to have any number of parameters, which means that the convolutional kernel is no longer limited to the standard square network, but can adopt more diverse and flexible shapes according to image characteristics and task requirements. When working with different images and targets, AKConv’s convolution kernel is able to automatically adjust its sampling shape. A new coordinate generation algorithm is introduced to generate initial sampling coordinates for convolutional kernels of varying sizes and shapes. Figure 4 shows the schematic structure of AKConv (Adapted from 32), and the three rows at the bottom show the changes in the sampling coordinates, which are the initial sampling position of the convolutional kernel without any offset, the learned offset that will be applied to the original coordinates, and the sampling coordinates after the offset is applied.

The sampling network used by the traditional convolution operation is regular, while AKConv aims at the irregularly shaped convolutional kernel, so an arbitrary-size convolution algorithm is designed, which can generate the initial sampling coordinates of the convolution kernel ${P}_{n}$. The process first generates a regular sampling grid, then creates an irregular grid for the rest of the sampling points, and finally stitches the two parts of the grid into a complete sampling grid. In the algorithm, the point in the upper left corner (0,0) is used as the sampling origin. After the initial coordinates ${P}_{n}$ of the irregular convolution are defined, the convolution operation corresponding to the ${P}_{0}$ position is defined as follows Eq. (1).

1 $$Conv\left( {P_{0} } \right) = \sum \omega \times \left( {P_{n} + P_{0} } \right)$$

where ω represents the convolution parameter. By replacing standard convolutions with AKConv in the neck, the model adapts kernel shapes to irregularly sized targets (e.g., windproof locating rings), improving small-object detection accuracy without increasing parameters.

## Experimental setup

### Experimental configuration

The experimental algorithms were implemented in Python 3.9 under the PyTorch 2.0.1 deep learning framework, using PyCharm as the integrated development environment. Network model training and related work were conducted on an NVIDIA GeForce RTX 4080 GPU.

### Self-constructed catenary dataset

**Fig. 5:**
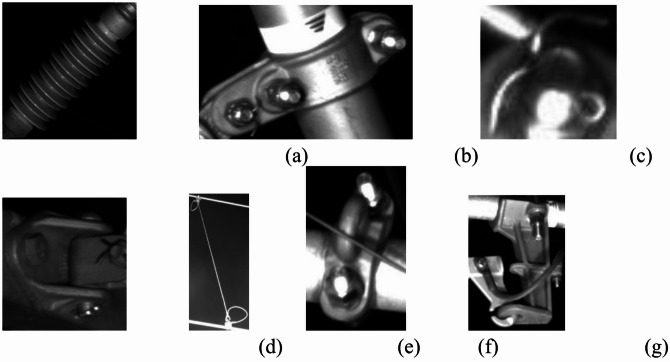
Dataset label categories.

The image data used in the experiment is the data obtained from the inspection of the high-speed railway catenary of the Hohhot-Ulanqab section by the high-speed rail comprehensive inspection vehicle. A total of 10,043 images were manually annotated by labelimg. We randomly split the dataset into a training set (8034 images, 80%) and a validation set (2009 images, 20%). The random splitting ensures that the training and validation sets are from the same distribution and helps to avoid bias. The dataset format was YOLO format, and the detected label categories are shown in Fig. 5.

For model training, images were resized to 640 × 640 pixels using bilinear interpolation and normalized using ImageNet standards (mean = [0.485, 0.456, 0.406], std = [0.229, 0.224, 0.225]).

It is worth noting that, despite the growing number of studies on deep learning-based detection of high-speed railway catenary components, publicly available standardized datasets remain scarce. Most existing research relies on self-collected datasets that are often not released, which hinders fair comparison and reproducibility of methods. In this work, we have publicly released our self-constructed catenary component dataset on the AI Studio platform (https://aistudio.baidu.com/datasetdetail/343828).This initiative aims to facilitate future comparative studies and contribute to the standardization and openness of data in this research field. Although a single random split was used due to computational constraints, the dataset was stratified to preserve the distribution of object categories and sizes. Future work will include cross-validation to further enhance robustness.

**Fig. 6:**
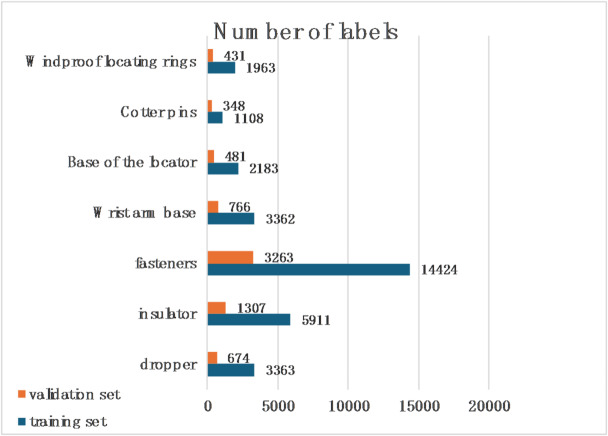
Number of labels in the training and validation sets.

Figure 6 shows the distribution of labels across categories in the training and validation sets. Small objects (area < 322 pixels) accounted for approximately 37.2% of all instances, primarily consisting of fasteners, cotter pins, and windproof locating rings.

### Public dataset validation: CPLID

**Fig. 7:**
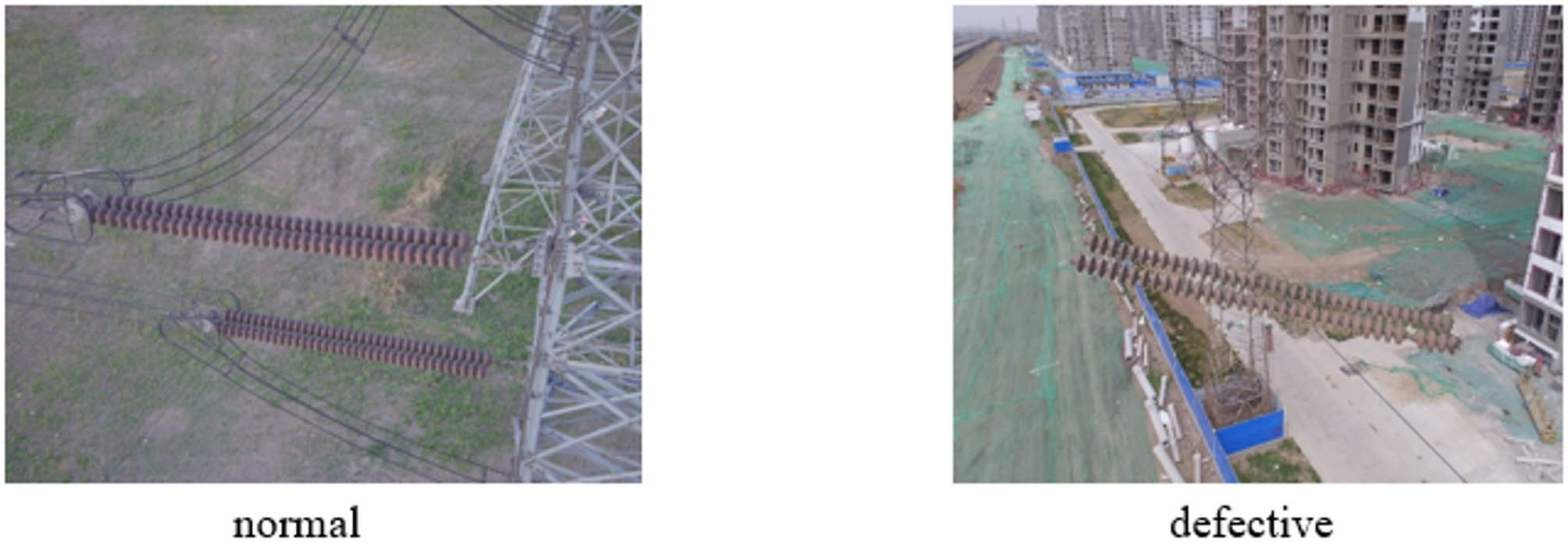
CPLID dataset.

To validate the generalization capability of our method, we conducted additional experiments on the publicly available CPLID (Chinese Power Line Insulator Dataset)^33^. This dataset contains 848 aerial insulator images with the following characteristics: 600 normal insulator images with bounding box annotations; 248 defective insulator images with dual annotations for insulator regions and defect areas. The two categories are shown in Fig. 7.

Data Processing: We processed the CPLID dataset into a unified format for insulator condition classification. Insulator instances were labeled as “normal” or “defective” based on the presence of defect regions within insulator bounding boxes (IoU threshold: 0.3). The dataset was split into training (80%) and validation (20%) sets using stratified sampling.

Evaluation Protocol: All models were trained and evaluated on CPLID using the same configuration as our main experiments to ensure fair comparison.

### Evaluate metrics

Considering that MSIM-Yolov11m is an improved algorithm based on YOLOv11m, to evaluate the detection effect of the catenary component detection model, the accuracy $P$ (Precision), recall rate $R$(Recall),$AP$ (Average Precision) and $mAP$(mean average precision) are still used as the evaluation indexes, and the specific expressions are as follows:

2 $$P = \frac{{T_{P} }}{{T_{P} + F_{P} }}$$

3 $$R = \frac{{T_{P} }}{{T_{P} + F_{N} }}$$

4 $$AP = \mathop \smallint \limits_{0}^{1} PdR$$

5 $$mAP = \frac{{\mathop \sum \nolimits_{i}^{n} AP\left( i \right)}}{n}$$

Among them, $P$ is the proportion of true positive samples in the predicted positive samples, and $R$ is the proportion of correctly predicted positive samples in the total positive samples;${ T}_{P}$ (True Positive) is a positive example of a correct prediction; ${F}_{P}$(False Positive) is a negative example that is incorrectly predicted as a positive example by the model; ${F}_{N}$ (False Negative) is a positive example that is incorrectly predicted as a negative example by the model; The $AP$ value refers to the area enclosed by the $P$-$R$ curve and the coordinate axis; $mAP$ is an important indicator to measure the detection accuracy in object detection, representing the average $AP$ of each category, the larger the value of $mAP$, the better the detection effect of the algorithm, and the higher the recognition accuracy.

Additionally, we measure frames per second (FPS) and floating point operations (FLOPs) to assess real-time capability and computational efficiency. FPS quantifies the number of images processed per second, tested on an NVIDIA GeForce RTX 4080 GPU with a batch size of 8. FLOPs represent the total floating-point operations required for a single forward pass, calculated at an input resolution of 640 × 640. Higher FPS implies stronger real-time performance, while lower FLOPs indicate better suitability for resource-constrained environments.

Following COCO evaluation standards, we defined small objects as those with an area less than 32^2^ pixels, and report AP_s_ for comprehensive evaluation.

## Analysis of experimental results

### Comparative experiments

For the dataset constructed in this paper, a variety of models and the MSIM-Yolov11m model in this paper are used to compare the detection effect of catenary parts. In this model training, the input image size is set to 640 × 640, the learning rate is set to 0.01, the threshold of the IOU of the regional recommendation network is set to 0.7, the batch size is set to 8, the optimization method is SGD, the momentum parameter is set to 0.937.

Our comparative analysis includes models that represent key developments in real-time object detection. While we recognize the value of broader comparisons, our selection focuses on models most relevant to our target application domain: SSD provides a baseline from the earlier generation of single-stage detectors, YOLO series (v8m, v9m, v10b, v11m) represent the state-of-the-art in real-time detection and serve as direct architectural comparisons. We specifically highlight comparison with YOLOv9m** as it represents a high-accuracy baseline, against which we demonstrate significant efficiency improvements. The performance metrics of the compared models are shown in Table 1.

**Table 1:** Experimental results comparison of different models.

Network model	P/%	R/%	mAP50-95/%	APs/%	Params/M	FLOPs/G	FPS
SSD	89.8	/	55.3	42.1	24.4	61.3	/
YOLOv8m	95	97.1	76	64.9	25.8	78.9	238
YOLOv9m	95.3	96.6	78.3	68	32.7	131.5	116.3
YOLOv10b	93.9	95.6	72.9	61.4	20.4	92	192.3
YOLOv11m	95	96.4	75	63.4	20	68	227.3
MSIM-Yolov11m	96.4	96.3	78.3	67.8	17.9	65.1	196.1

The selection of comparison models—SSD, YOLOv8m, YOLOv9m, and YOLOv10b—is driven by their representativeness in balancing real-time performance and accuracy. SSD provides a lightweight baseline for real-time applications. YOLOv9m emphasizes high precision through deeper networks, while YOLOv8m optimizes the speed-accuracy trade-off. YOLOv10b incorporates advanced designs like task-aligned learning, underscoring the competitiveness of our multi-scale interaction module (MSIM) and adaptive attention.

### Small target detection performance

In the high-speed rail catenary parts, the commonly used patter pins, bolts and other parts are usually small in size, and it is difficult to achieve good detection results, and the data set used in this paper includes fasteners, cotter pins and windproof positioning rings, which belong to the category of small labels, and the detection effect of these three types of small targets is more obvious while improving the overall detection effect, as shown in Table 2.

**Table 2:** Comparison of small target detection results.

Network model	Detection site	mAP50-95/%	Precision/%	Recall/%
YOLOv11m	fasteners	67.2	96.9	97.5
	Cotter pins	64	92.3	89.8
	Windproof locating rings	59.2	97.2	97.2
MSIM-Yolov11m	fasteners	71.3	97.8	97.7
	Cotter pins	67.3	94.3	89.7
	Windproof locating rings	64.7	97.9	97.4

As shown in the results in the table, the MSIM-Yolov11m achieves better results in small target detection, with the fastener, cotter pin, and windproof locating ring increasing by 4.1%, 3.3%, and 5.5% on the mAP50-95 values, respectively.

### Ablation experiments

The MSIM-Yolov11m high-speed rail catenary component detection model was tested to verify the effectiveness of the LSKA module, BiFPN module and AKConv convolution method, and Table 3 shows the results of network model detection under different improvement strategies in ablation experiments.

**Table 3:** Results of ablation experiments.

Network model	LSKA	BiFPN	AKConv	mAP50-95/%		Params/M	FLOPs/G	FPS
YOLOv11m	×	×	×	75	20		68	227.3
YOLOv11m-L	√	×	×	77.6	19.8		67.5	222.2
YOLOv11m-B	×	√	×	77.8	20		67.7	217.4
YOLOv11m-A	×	×	√	75.4	18.1		65.2	192.3
YOLOv11m-LB	√	√	×	78.1	19.8		67.5	212.7
YOLOv11m-LA	√	×	√	77.9	17.9		65.1	294.1
YOLOv11m-BA	×	√	√	77.6	18.1		65.2	188.6
MSIM-Yolov11m	√	√	√	78.3	17.9		65.1	196.1

Comparing Table 3, it can be seen that the LSKA module, BiFPN module, and AKConv are helpful to improve the detection performance of the original model. Specifically, the attention layer in the C2PSA module of the YOLOv11m model was replaced with LSKA and combined into a C2PSA_LSKA module, and the feature extraction ability of the C2PSA module was enhanced by using the detached convolutional kernel characteristics of LSKA The detection effect of small targets was improved, and the mAP50-95 value was increased by 2.6%; By adding BiFPN to the concat layer as a concat_BiFPN layer, BiFPN can make full use of feature information at different scales through bottom-up feature fusion and top-down feature enhancement, thereby improving the accuracy of target detection, and the mAP50-95 value is increased by 2.8%. After replacing the convolution method in the neck layer of the original model with AKConv, the number of model parameters is further reduced because it can provide any number of parameters and arbitrary sampling shapes for the convolution kernel, and the mAP50-95 value is increased by 0.4%. Compared with the original model, although the frames per second (FPS) of the detected image in this algorithm are reduced, the detection accuracy is improved. The combination experiments show that LSKA and BiFPN have the most significant complementary effects, together contributing a 3.1% improvement in mAP50-95. While AKConv provides modest improvements alone, it contributes to parameter reduction and enhances performance when combined with other modules.

### Cross-dataset validation on CPLID

To evaluate generalization capability, we conducted experiments on the public CPLID dataset. Table 4 shows the comparative results.

**Table 4:** Experimental results on CPLID dataset.

Network model	mAP50-95/%	Precision/%	Recall/%
YOLOv8m	86.1	95	95.4
YOLOv9m	87	95.5	96.2
YOLOv11m	86.4	94.2	94.3
MSIM-Yolov11m	87.5	95.4	97

The results on the CPLID dataset demonstrate that our method achieves the best performance while maintaining efficiency, indicating good generalization to public datasets.

## Conclusions

In this paper, we presented MSIM-YOLOv11m, a novel object detection model tailored for high-speed railway catenary defect detection. Our work goes beyond simply combining existing modules; it provides a systematic integration of LSKA, BiFPN, and AKConv, each addressing specific challenges in the catenary inspection task. The LSKA module enhances the attention on small and critical parts, the BiFPN module effectively fuses features across different scales to handle the size variation of components, and the AKConv module adapts to the irregular shapes of objects like windproof locating rings. This cohesive design leads to a model that not only achieves high accuracy (78.3% mAP50-95 on our self-constructed dataset) but also reduces the computational cost by 50.5% compared to YOLOv9m, demonstrating a significant step towards practical deployment.

Furthermore, the model’s generalization capability was validated on the public CPLID dataset, where it achieved a mAP50-95 of 87.5%, outperforming other YOLO variants. This result confirms the robustness and adaptability of our approach across different datasets and environmental conditions.

However, the study has several limitations. First, although cross-dataset validation was conducted, the model was primarily trained and validated on a self-collected dataset, which may still limit its generalizability to other railway environments. Second, due to technical constraints, we have not yet deployed and validated the model on actual edge devices, which is crucial for assessing its real-world applicability in inspection systems.

In future work, we plan to: (1) extend the dataset to include more fault categories and environmental variations; (2) explore collaboration opportunities to deploy the model on embedded platforms for real-time inference tests; (3) explore end-to-end recognition of defects beyond mere component detection, such as crack identification and wear assessment. Furthermore, although the current comparative study focuses on the most representative real-time detectors (e.g., the YOLO series), future work will include extensive comparisons with transformer-based models (e.g., DETR and its variants) and other specialized lightweight detectors to further position the performance of our method within the broader landscape of object detection research.

## Data Availability

The datasets generated and/or analysed during the current study are available in the https://aistudio.baidu.com/datasetdetail/343828.
